# Cardiac arrest secondary to mitral annular disjunction: a case report

**DOI:** 10.1093/ehjcr/ytag355

**Published:** 2026-05-26

**Authors:** Ahmed Smman, Jacques-Henri Meurgey, Raj Khiani, Nelson Amaral

**Affiliations:** Cardiology, Royal Free London NHS Foundation Trust, Royal Free Hospital, Pond Street, London NW3 2QG, United Kingdom; Cardiology, Royal Free London NHS Foundation Trust, Royal Free Hospital, Pond Street, London NW3 2QG, United Kingdom; Cardiology, Royal Free London NHS Foundation Trust, Royal Free Hospital, Pond Street, London NW3 2QG, United Kingdom; Cardiology, University College London Faculty of Medical Sciences, 74 Huntley Street, London WC1E 6BT, United Kingdom; Cardiology, Royal Free London NHS Foundation Trust, Royal Free Hospital, Pond Street, London NW3 2QG, United Kingdom; Cardiology, University College London Faculty of Medical Sciences, 74 Huntley Street, London WC1E 6BT, United Kingdom

**Keywords:** mitral valve prolapse, Valvular heart disease, Cardiac arrest, Case report

## Abstract

**Background:**

Mitral annular disjunction with bileaflet mitral valve prolapse is increasingly recognized as a substrate for malignant ventricular arrhythmia and sudden cardiac arrest. Prior myocardial fibrosis adjacent to papillary muscles and frequent ventricular ectopy increase arrhythmic risk.

**Case Summary:**

We describe the case of a woman in her 40s who suffered an out-of-hospital ventricular fibrillation cardiac arrest with immediate bystander CPR and ambulance arrival within 5 min. Return of spontaneous circulation occurred approximately 30 minutes after multiple shocks. She had a background of bileaflet mitral valve prolapse discovered on routine investigation following a syncopal episode in her youth. She was known to have significant mitral annular disjunction and focal mid-wall/subepicardial late gadolinium enhancement on serial cardiac MRI. An implantable loop recorder previously demonstrated frequent multifocal ventricular ectopy but had reached end-of-life by the time of the case. Subsequent testing excluded coronary disease, pulmonary embolus, and long QT as causes for her arrest. A transvenous implantable cardioverter defibrillator was inserted for secondary prevention and pacing capability should she require it, with mitral valve replacement following this.

**Discussion:**

This case exemplifies mitral annular disjunction arrhythmic syndrome presenting as ventricular fibrillation arrest in a patient with bileaflet prolapse, focal myocardial fibrosis, and frequent ventricular ectopy, managed with ICD implantation and eventual surgical valve replacement. Current consensus would have graded her arrhythmic risk as intermediate.

Learning Points

- Mitral annular disjunction and mitral valve prolapse are established arrhythmic substrates often overlooked in the investigation of cardiac arrest.
- Risk stratification can be augmented with multimodality imaging (CMR and TTE/TOE) and rhythm monitoring.
- Prophylactic ICD and/or surgery should be considered for intermediate- and high-risk patients.

## Introduction

Mitral Annular Disjunction (MAD) is defined by the separation of the mitral valve annulus during systole, where the left ventricle (LV) contracts and the mitral valve (MV) annulus slides and detaches from the myocardium.^1^ Conditions that predispose towards MAD include a myxomatous mitral valve, mitral valve prolapse (MVP), mitral valve degeneration, genetic and connective tissue diseases.^2,3^ MAD has a prevalence of around 2% and can be benign in isolation, but is seen in up to 55% of patients with mitral valve prolapse.^4^ MAD has a strong association with ventricular arrhythmias, with up to 15% of unexplained cardiac arrests associated with MAD.^1^ MAD is generally diagnosed with transthoracic (TTE) or transoesophageal echocardiography (TOE). Still, CMR can also help to characterize the degree of myocardial and papillary muscle fibrosis.

## Summary figure

**Figure:**
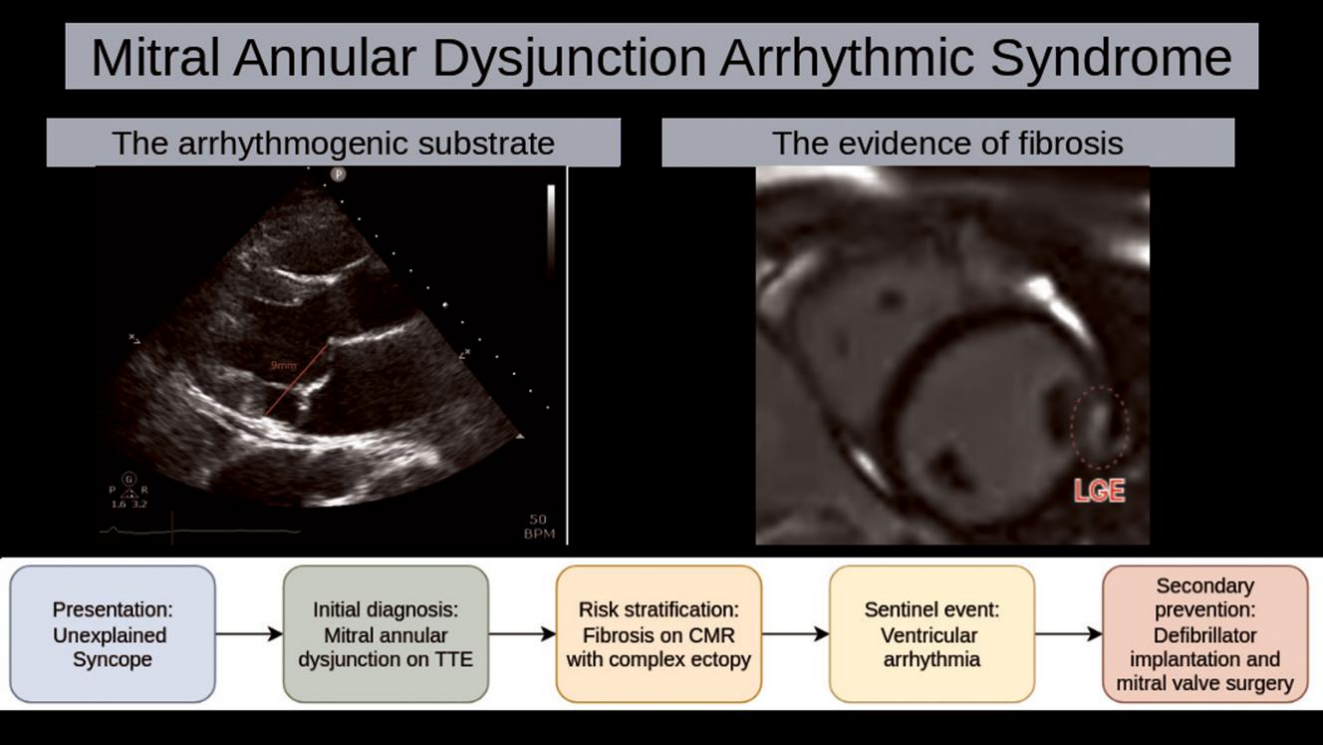


## Case description

A woman in her 40s collapsed following an agonal gasp. Her children alerted their neighbour, who initiated bystander cardiopulmonary resuscitation (CPR) and called emergency services, who arrived within 4 min. The initial rhythm was ventricular fibrillation (VF). She had approximately 30 min of downtime with five shocks before return of spontaneous circulation (ROSC). Post-ROSC ECG demonstrated sinus rhythm with bigeminy in a posterior papillary muscle morphology with precordial transition at V2 and a coupling interval of 480 ms.

Post-ROSC, she was hypoxic, minimally responsive with a Glasgow Coma Scale of 6/15 and requiring inotropes to maintain stable blood pressure, so she was intubated and transferred to intensive care. Cardiovascular examination was reportedly unremarkable. ECG 2 days later again demonstrated sinus with trigeminy in a posterolateral morphology with precordial transition at V2 and a coupling interval of 280 ms. Retrospective review of her ECGs demonstrated daytime predominance of her premature ventricular complexes (PVCs).

Laboratory blood results, including full blood count, creatinine, electrolytes, thyroid function, ferritin, and CRP, were within normal limits with a mildly elevated but static high-sensitivity troponin at 73. CT coronary angiogram (CTCA) excluded spontaneous coronary artery dissection (SCAD) or coronary artery disease as the cause for the cardiac arrest.

TTE showed normal LV size but impaired systolic function at 40% and a dyskinetic septum, but no other regional wall movement abnormalities. The mitral valve demonstrated bi-leaflet MVP with mild-to-moderate late systolic MR (visually assessed), as well as moderate tricuspid regurgitation.

She had a past medical history of vagal syncope at age 18, with bileaflet MVP discovered incidentally on subsequent TTE. Cardiac MRI (CMR) at that time showed a normal left ventricular ejection fraction (LVEF) but a small focus of mid-anterolateral subepicardial fibrosis/late gadolinium enhancement (LGE). She also suffered from recurrent palpitations for which she had an implantable loop recorder (ILR) *in situ*. Interrogation of the device 4 years before her arrest demonstrated a 7.8% multifocal PVC burden with triplets, with a subsequent interrogation showing only ‘rare’ PVCs once started on bisoprolol. Due to her response to bisoprolol, a repeat ILR was not inserted at device end-of-life. Holter monitor 2 years before her arrest showed sinus rhythm with 13% multifocal PVC burden and occasional dropped beats (Wenckebach), but no incidence of non-sustained ventricular tachycardia or atrial fibrillation. Unfortunately, her ILR could not be interrogated on this admission due to battery depletion. Her only regular medication was bisoprolol 2.5 mg twice daily to suppress PVCs.

CMR in the year before admission showed a dilated atrium (43.5 mL/m^2^) and mitral valve annulus, bileaflet MVP, and a cleft between P1 and P2. The annular disjunction distance measured 9 mm with late systolic MR. There was now mid-wall LGE of the mid-inferolateral LV, adjacent to the anterolateral papillary muscle, involving approximately 5% of the myocardium by mass. An exercise ECG was inconclusive as it was prematurely terminated due to dizziness.

She had no family history of coronary artery disease, cardiomyopathies, or sudden cardiac death. She was independent of her activities of daily living and did not smoke, drink alcohol, or use recreational drugs.

On further questioning, she described 6 months of subjective fatigue, without chest pain or breathlessness, and swimming frequently with good exercise tolerance. She had ongoing palpitations and dizziness at this time but had had no syncopal episodes.

She remained in intensive care for 4 days and was weaned off inotropes and vasopressors and was extubated, with her stay complicated by aspiration pneumonia. Telemetry showed sinus bradycardia (with no AV block) with frequent ventricular ectopics, and amiodarone was commenced to suppress this. She was stepped down to the coronary care unit on day 5, whereupon her bisoprolol was up-titrated, and amiodarone was stopped due to QTc prolongation (492 ms).

**Figure 1:**
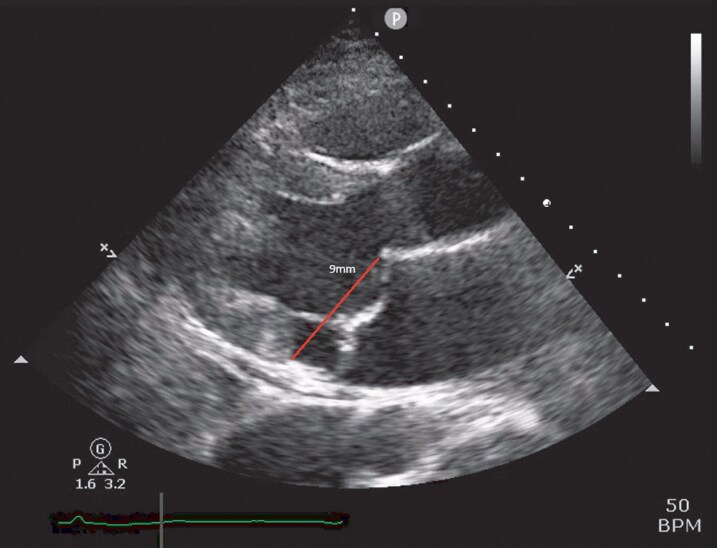
Parasternal long-axis view in mid-systole showing mitral annular dysjunction distance of 9 mm.

**Figure 2:**
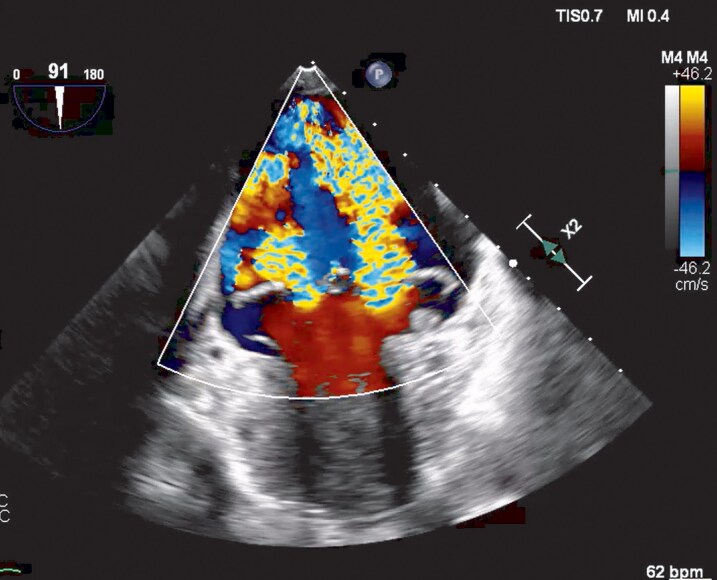
Midoesophageal two-chamber view showing two jets of MR in mid-systole.

**Figure 3:**
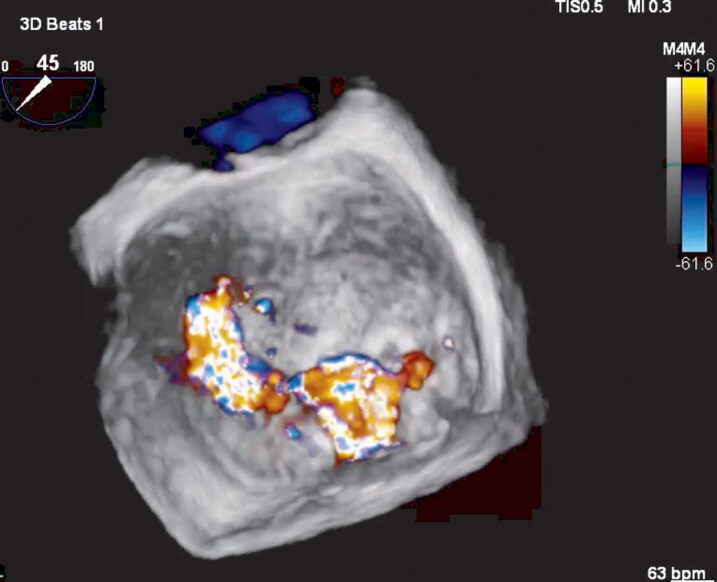
Mid-oesophageal 3D echo view showing two jets in mid-systole.

The TOE following discharge from intensive care demonstrated a myxomatous MV with bileaflet prolapse involving all scallops except P3, with a cleft between P1 and P2. There was mitral annular disjunction measuring 10–12 mm at systole with a dilated mitral annulus (*Figure 1*). Her mitral regurgitation was, in fact, moderate-to-severe with a regurgitant orifice area of 0.34 cm2, proximal isovelocity surface area of 1 cm, vena contracta width of 0.7 cm. There were 2 jets, the larger measuring 49 mL and the smaller jet unquantifiable, suggestive of severe MR (*Figures 2 and 3*). The tricuspid valve also demonstrated bileaflet prolapse with mild-moderate regurgitation, though estimated pulmonary artery systolic pressure was only 14 mmHg. All other structures were within normal limits. The initial TTE was likely under-reported due to a combination of limited views on intensive care, post-arrest haemodynamics, a late systolic jet, and the presence of multiple jets better visualized on TOE.

Following consensus discussion, she underwent secondary prevention cardioverter-defibrillator (ICD) implantation, with a transvenous approach due to previous bradycardia and AV block on Holter, should she require (biventricular) pacing in the future. She suffered from persistent cognitive and memory dysfunction following her arrest and intensive care stay. Her mitral valve replacement was delayed to allow her a more complete neurological recovery due to concerns regarding the effect cardiopulmonary bypass may have on this, as her exercise tolerance remained unaffected. Catheter ablation was not pursued; there is no documentation of electrophysiological evaluation, and she did not undergo intra-operative ablation.

Her relatives were screened for MVP with TTE and ECG. She subsequently underwent testing for Long QT syndrome (LQTS) and was not a carrier of a known mutation. She has returned to work and has recorded no ventricular arrhythmias on her most recent device check.

## Discussion

The diagnosis in this case is Mitral Annular Disjunction Arrhythmic Syndrome, a condition characterized by ventricular arrhythmias and sudden cardiac death in patients with MAD.

The pathophysiology of MAD centres on the disruption of the normal fibrous continuity at the base of the MV. This causes systolic traction on the inferolateral wall adjacent to the MV and leads to biomechanical stretch on the myocardium, causing myxomatous degeneration and disruption in annular-ventricular conduction. PVC-triggered VF is thought to be the main mechanism of SCD in this cohort.^5^

Evaluation of MAD involves TTE to assess MV morphology and annular integrity. TOE is recommended when TTE is equivocal. CMR can identify areas of LGE (fibrosis) to prognosticate arrhythmic and sudden cardiac death risk.^3,5^ Despite surgical correction of valvular insufficiency, residual arrhythmic risk may persist in patients with extensive MAD.^6^

Essayagh *et al*. reviewed a series of case reports spanning over 20 years, demonstrating that patients with MVP without degenerative mitral regurgitation have a normal life expectancy.^5^ Asymptomatic patients with MVP with severe MR had twice the baseline population risk of sudden cardiac death. Bileaflet MVP was associated with higher arrhythmia risk, but no significant impact on mortality. This is echoed in the 2022 EHRA consensus guidelines on arrhythmic mitral valve disorders.^7^

As this patient had had no significant arrhythmic events before her collapse, she would have been deemed intermediate risk. However, she displays a number of features that are known to carry increased ventricular arrhythmia risk: Young age, female sex, her significant mitral annular dysjunction at 9–12 mm, bileaflet MVP,^8^ focal mid-wall LGE^5,9^ and high burden of ventricular ectopy with daytime predominance and early precordial transition.^10^

Treatment of MAD depends on disease severity. MAD does not seem to impact the feasibility and quality of valve repair; decisions surrounding repair or replacement can be taken based on conventional parameters for mitral regurgitation (MR).^2,4^

Other causes of arrhythmic cardiac arrest, such as LQTS should be considered in such patients. Although her CT coronary angiogram excluded a SCAD or ischaemic cause, coronary assessment with either a CTCA or invasive coronary angiogram should be considered for any patient with an unexplained cardiac arrest.

## Data Availability

All relevant data have been included in the text. Further anonymized data. The data underlying this article will be shared on reasonable request to the corresponding author.

## Lead author biography

Dr Ahmed Smman is a Senior Clinical Fellow in Cardiology at Barnet Hospital, part of the Royal Free London NHS Foundation Trust. He graduated from Alexandria University, Egypt, in 2017 and obtained his Master’s degree in Cardiology in 2023. His clinical interests include cardiac electrophysiology and cardiac rhythm management. He has contributed to original research published in the Egyptian Heart Failure Journal and has led quality improvement initiatives within his department.

## Notes

**Consent:** The authors have obtained written consent from the patient to publish this case report.
